# Supplementary material for: Modeling glioblastoma heterogeneity as a dynamic network of cell states
Source: Mol Syst Biol. 2021 Sep 16;17(9):e10105. doi: 10.15252/msb.202010105 (PMC8444284; doi:10.15252/msb.202010105)
Supplement: Supplementary file 5 — Source Data for Figure 3 [file MSB-17-e10105-s001.zip › Figure3A_sourcedata/GSEA_3065/nosigdb_state5.GseaPreranked.1622718435914/DOROSZKO_U3180MG_CLUSTER_4.html]

Details for gene set DOROSZKO\_U3180MG\_CLUSTER\_4[GSEA]

|  || Dataset | state5 |
| Phenotype | NoPhenotypeAvailable |
| Upregulated in class | na\_neg |
| GeneSet | DOROSZKO\_U3180MG\_CLUSTER\_4 |
| Enrichment Score (ES) | -0.5365263 |
| Normalized Enrichment Score (NES) | -1.9509295 |
| Nominal p-value | 0.0 |
| FDR q-value | 2.633059E-4 |
| FWER p-Value | 0.005 |
Table: GSEA Results Summary

  

Fig 1: Enrichment plot: DOROSZKO\_U3180MG\_CLUSTER\_4      
 Profile of the Running ES Score & Positions of GeneSet Members on the Rank Ordered List

  

| PROBE | GENE SYMBOL | GENE\_TITLE | RANK IN GENE LIST | RANK METRIC SCORE | RUNNING ES | CORE ENRICHMENT || 1 | CNIH4 |  |  | 169 | 0.465 | -0.0062 | No |
| 2 | PDGFRA |  |  | 175 | 0.460 | 0.0060 | No |
| 3 | LBR |  |  | 198 | 0.440 | 0.0157 | No |
| 4 | ARL6IP1 |  |  | 232 | 0.418 | 0.0236 | No |
| 5 | EMP3 |  |  | 264 | 0.400 | 0.0312 | No |
| 6 | PIF1 |  |  | 266 | 0.400 | 0.0422 | No |
| 7 | SUGP2 |  |  | 278 | 0.395 | 0.0519 | No |
| 8 | HSPA13 |  |  | 511 | 0.307 | 0.0342 | No |
| 9 | TXNDC12 |  |  | 527 | 0.304 | 0.0409 | No |
| 10 | FKBP2 |  |  | 672 | 0.270 | 0.0321 | No |
| 11 | IDH1 |  |  | 793 | 0.242 | 0.0252 | No |
| 12 | SUN2 |  |  | 829 | 0.236 | 0.0278 | No |
| 13 | HSD17B11 |  |  | 949 | 0.219 | 0.0204 | No |
| 14 | TMED5 |  |  | 1054 | 0.204 | 0.0142 | No |
| 15 | PPP1R35 |  |  | 1368 | 0.164 | -0.0167 | No |
| 16 | RBMX |  |  | 1512 | 0.151 | -0.0287 | No |
| 17 | GPSM2 |  |  | 1612 | 0.141 | -0.0360 | No |
| 18 | REEP4 |  |  | 1793 | 0.127 | -0.0529 | No |
| 19 | KIF20B |  |  | 1962 | 0.114 | -0.0688 | No |
| 20 | TMEM19 |  |  | 2286 | 0.093 | -0.1028 | No |
| 21 | NDE1 |  |  | 2625 | 0.072 | -0.1391 | No |
| 22 | PCBD2 |  |  | 2684 | 0.069 | -0.1438 | No |
| 23 | CCDC18 |  |  | 2814 | 0.062 | -0.1567 | No |
| 24 | SEC22C |  |  | 2854 | 0.060 | -0.1594 | No |
| 25 | MEX3A |  |  | 2917 | 0.057 | -0.1649 | No |
| 26 | SRRT |  |  | 2930 | 0.056 | -0.1647 | No |
| 27 | HP1BP3 |  |  | 3122 | 0.046 | -0.1851 | No |
| 28 | G2E3 |  |  | 3493 | 0.028 | -0.2263 | No |
| 29 | GAS2L3 |  |  | 3714 | 0.020 | -0.2507 | No |
| 30 | FUBP1 |  |  | 3894 | 0.012 | -0.2706 | No |
| 31 | DLGAP5 |  |  | 3907 | 0.011 | -0.2717 | No |
| 32 | NCAPD2 |  |  | 3914 | 0.011 | -0.2721 | No |
| 33 | NUP37 |  |  | 3930 | 0.010 | -0.2735 | No |
| 34 | AC026401.3 |  |  | 4096 | 0.003 | -0.2921 | No |
| 35 | IMMP1L |  |  | 4163 | 0.000 | -0.2996 | No |
| 36 | KIAA0586 |  |  | 4229 | -0.002 | -0.3069 | No |
| 37 | TNFAIP8L1 |  |  | 4236 | -0.003 | -0.3075 | No |
| 38 | LMO7 |  |  | 4238 | -0.003 | -0.3075 | No |
| 39 | SAPCD2 |  |  | 4286 | -0.005 | -0.3127 | No |
| 40 | EMC9 |  |  | 4405 | -0.010 | -0.3258 | No |
| 41 | CEP70 |  |  | 4512 | -0.014 | -0.3374 | No |
| 42 | CKAP5 |  |  | 4519 | -0.015 | -0.3377 | No |
| 43 | DCP2 |  |  | 4575 | -0.017 | -0.3435 | No |
| 44 | KIF14 |  |  | 4758 | -0.024 | -0.3634 | No |
| 45 | CENPE |  |  | 4787 | -0.025 | -0.3659 | No |
| 46 | PRR11 |  |  | 4822 | -0.026 | -0.3690 | No |
| 47 | HMMR |  |  | 4826 | -0.027 | -0.3686 | No |
| 48 | TROAP |  |  | 4869 | -0.028 | -0.3726 | No |
| 49 | KIF18A |  |  | 4962 | -0.032 | -0.3821 | No |
| 50 | USP13 |  |  | 5088 | -0.037 | -0.3953 | No |
| 51 | CCNB1 |  |  | 5111 | -0.037 | -0.3968 | No |
| 52 | KATNA1 |  |  | 5142 | -0.038 | -0.3991 | No |
| 53 | PRMT5 |  |  | 5155 | -0.039 | -0.3994 | No |
| 54 | EEF1AKMT2 |  |  | 5162 | -0.039 | -0.3990 | No |
| 55 | BUB1 |  |  | 5195 | -0.040 | -0.4015 | No |
| 56 | SNAPC1 |  |  | 5218 | -0.041 | -0.4029 | No |
| 57 | ODF2 |  |  | 5219 | -0.041 | -0.4017 | No |
| 58 | SPAG5 |  |  | 5225 | -0.041 | -0.4011 | No |
| 59 | ECT2 |  |  | 5264 | -0.043 | -0.4043 | No |
| 60 | PPP2R5C |  |  | 5373 | -0.048 | -0.4152 | No |
| 61 | CCNB2 |  |  | 5382 | -0.048 | -0.4148 | No |
| 62 | HNRNPA1P48 |  |  | 5428 | -0.049 | -0.4185 | No |
| 63 | NEK2 |  |  | 5442 | -0.050 | -0.4186 | No |
| 64 | SPDL1 |  |  | 5460 | -0.051 | -0.4191 | No |
| 65 | FAM83D |  |  | 5467 | -0.051 | -0.4184 | No |
| 66 | DAZAP1 |  |  | 5486 | -0.052 | -0.4189 | No |
| 67 | RANGRF |  |  | 5552 | -0.055 | -0.4248 | No |
| 68 | TTK |  |  | 5604 | -0.058 | -0.4290 | No |
| 69 | RNF26 |  |  | 5657 | -0.060 | -0.4332 | No |
| 70 | SPA17 |  |  | 5739 | -0.063 | -0.4406 | No |
| 71 | KIF11 |  |  | 5795 | -0.065 | -0.4450 | No |
| 72 | CDCA8 |  |  | 5863 | -0.068 | -0.4507 | No |
| 73 | KNSTRN |  |  | 5923 | -0.070 | -0.4555 | No |
| 74 | RANGAP1 |  |  | 6212 | -0.086 | -0.4858 | No |
| 75 | RUVBL2 |  |  | 6371 | -0.093 | -0.5011 | No |
| 76 | CENPA |  |  | 6374 | -0.094 | -0.4987 | No |
| 77 | AIMP2 |  |  | 6494 | -0.101 | -0.5094 | No |
| 78 | PLK1 |  |  | 6581 | -0.107 | -0.5162 | No |
| 79 | ZNF106 |  |  | 6693 | -0.113 | -0.5256 | No |
| 80 | CEP55 |  |  | 6700 | -0.114 | -0.5231 | No |
| 81 | RHNO1 |  |  | 6734 | -0.116 | -0.5236 | No |
| 82 | NUDT11 |  |  | 6758 | -0.118 | -0.5230 | No |
| 83 | ETV4 |  |  | 6761 | -0.118 | -0.5199 | No |
| 84 | ANLN |  |  | 6829 | -0.123 | -0.5241 | No |
| 85 | MAP4K4 |  |  | 6919 | -0.131 | -0.5306 | No |
| 86 | CRAT |  |  | 6922 | -0.131 | -0.5272 | No |
| 87 | CDC20 |  |  | 6985 | -0.136 | -0.5304 | No |
| 88 | AURKA |  |  | 6990 | -0.136 | -0.5271 | No |
| 89 | SEPHS1 |  |  | 7046 | -0.141 | -0.5294 | No |
| 90 | CDC25B |  |  | 7102 | -0.146 | -0.5316 | No |
| 91 | UBALD2 |  |  | 7137 | -0.149 | -0.5313 | No |
| 92 | ASF1A |  |  | 7155 | -0.151 | -0.5290 | No |
| 93 | SDCBP |  |  | 7222 | -0.158 | -0.5321 | Yes |
| 94 | TMEM160 |  |  | 7238 | -0.160 | -0.5294 | Yes |
| 95 | SMIM14 |  |  | 7281 | -0.165 | -0.5296 | Yes |
| 96 | EIF4E |  |  | 7292 | -0.166 | -0.5261 | Yes |
| 97 | SYNCRIP |  |  | 7350 | -0.175 | -0.5277 | Yes |
| 98 | HNRNPH3 |  |  | 7361 | -0.176 | -0.5239 | Yes |
| 99 | MDH1 |  |  | 7422 | -0.184 | -0.5256 | Yes |
| 100 | TIMM9 |  |  | 7435 | -0.185 | -0.5218 | Yes |
| 101 | ACTN1 |  |  | 7520 | -0.193 | -0.5260 | Yes |
| 102 | RUVBL1 |  |  | 7568 | -0.200 | -0.5258 | Yes |
| 103 | CDK2AP2 |  |  | 7640 | -0.209 | -0.5280 | Yes |
| 104 | CFAP36 |  |  | 7668 | -0.213 | -0.5252 | Yes |
| 105 | RNF5 |  |  | 7708 | -0.220 | -0.5235 | Yes |
| 106 | CDKN2D |  |  | 7721 | -0.222 | -0.5187 | Yes |
| 107 | MRPL47 |  |  | 7759 | -0.226 | -0.5166 | Yes |
| 108 | ECI2 |  |  | 7776 | -0.229 | -0.5120 | Yes |
| 109 | GDI2 |  |  | 7806 | -0.235 | -0.5088 | Yes |
| 110 | ARPP19 |  |  | 7811 | -0.236 | -0.5027 | Yes |
| 111 | MZT1 |  |  | 7838 | -0.242 | -0.4989 | Yes |
| 112 | PSMA4 |  |  | 7883 | -0.251 | -0.4969 | Yes |
| 113 | PSME2 |  |  | 7885 | -0.252 | -0.4900 | Yes |
| 114 | PRR13 |  |  | 7891 | -0.254 | -0.4835 | Yes |
| 115 | TMEM256 |  |  | 7924 | -0.261 | -0.4799 | Yes |
| 116 | HNRNPM |  |  | 7932 | -0.262 | -0.4734 | Yes |
| 117 | STIP1 |  |  | 7971 | -0.270 | -0.4702 | Yes |
| 118 | BZW1 |  |  | 7988 | -0.272 | -0.4645 | Yes |
| 119 | EZR |  |  | 8030 | -0.281 | -0.4613 | Yes |
| 120 | CCT8 |  |  | 8033 | -0.282 | -0.4537 | Yes |
| 121 | PPP1CC |  |  | 8035 | -0.282 | -0.4460 | Yes |
| 122 | FAM120AOS |  |  | 8078 | -0.293 | -0.4426 | Yes |
| 123 | CDKN2C |  |  | 8091 | -0.295 | -0.4358 | Yes |
| 124 | NUDCD2 |  |  | 8119 | -0.300 | -0.4305 | Yes |
| 125 | MORF4L2 |  |  | 8168 | -0.317 | -0.4271 | Yes |
| 126 | COX20 |  |  | 8194 | -0.325 | -0.4209 | Yes |
| 127 | PAIP1 |  |  | 8197 | -0.326 | -0.4121 | Yes |
| 128 | DDAH2 |  |  | 8204 | -0.328 | -0.4037 | Yes |
| 129 | ILF2 |  |  | 8220 | -0.331 | -0.3962 | Yes |
| 130 | CBX1 |  |  | 8222 | -0.331 | -0.3871 | Yes |
| 131 | ADI1 |  |  | 8226 | -0.332 | -0.3782 | Yes |
| 132 | RAB11A |  |  | 8228 | -0.332 | -0.3691 | Yes |
| 133 | IFT22 |  |  | 8246 | -0.337 | -0.3616 | Yes |
| 134 | AAMDC |  |  | 8258 | -0.341 | -0.3534 | Yes |
| 135 | LDHA |  |  | 8263 | -0.343 | -0.3443 | Yes |
| 136 | SSNA1 |  |  | 8276 | -0.347 | -0.3360 | Yes |
| 137 | KHDRBS1 |  |  | 8280 | -0.348 | -0.3267 | Yes |
| 138 | TPGS2 |  |  | 8295 | -0.353 | -0.3185 | Yes |
| 139 | GSPT1 |  |  | 8299 | -0.355 | -0.3089 | Yes |
| 140 | ANP32A |  |  | 8326 | -0.361 | -0.3019 | Yes |
| 141 | HDGF |  |  | 8330 | -0.362 | -0.2921 | Yes |
| 142 | CIB1 |  |  | 8354 | -0.371 | -0.2844 | Yes |
| 143 | RBM42 |  |  | 8367 | -0.376 | -0.2753 | Yes |
| 144 | GTF2A2 |  |  | 8390 | -0.385 | -0.2671 | Yes |
| 145 | MAZ |  |  | 8394 | -0.387 | -0.2567 | Yes |
| 146 | VBP1 |  |  | 8395 | -0.387 | -0.2460 | Yes |
| 147 | PLIN3 |  |  | 8403 | -0.388 | -0.2360 | Yes |
| 148 | CCT7 |  |  | 8416 | -0.392 | -0.2265 | Yes |
| 149 | PTTG1 |  |  | 8446 | -0.406 | -0.2185 | Yes |
| 150 | MAP1LC3B |  |  | 8463 | -0.416 | -0.2087 | Yes |
| 151 | TXNDC17 |  |  | 8485 | -0.428 | -0.1992 | Yes |
| 152 | NUCKS1 |  |  | 8502 | -0.435 | -0.1889 | Yes |
| 153 | ITGAE |  |  | 8507 | -0.438 | -0.1772 | Yes |
| 154 | SKA2 |  |  | 8515 | -0.442 | -0.1657 | Yes |
| 155 | CALM3 |  |  | 8517 | -0.443 | -0.1535 | Yes |
| 156 | NDUFA2 |  |  | 8575 | -0.472 | -0.1469 | Yes |
| 157 | RAB13 |  |  | 8590 | -0.481 | -0.1351 | Yes |
| 158 | COPRS |  |  | 8615 | -0.502 | -0.1239 | Yes |
| 159 | EIF4G2 |  |  | 8627 | -0.510 | -0.1110 | Yes |
| 160 | JPT1 |  |  | 8647 | -0.523 | -0.0986 | Yes |
| 161 | GLRX5 |  |  | 8685 | -0.548 | -0.0875 | Yes |
| 162 | DCTN3 |  |  | 8797 | -0.648 | -0.0821 | Yes |
| 163 | NDUFAF3 |  |  | 8845 | -0.704 | -0.0679 | Yes |
| 164 | UBE2S |  |  | 8901 | -0.772 | -0.0526 | Yes |
| 165 | TMSB10 |  |  | 8979 | -1.090 | -0.0311 | Yes |
| 166 | LGALS1 |  |  | 8982 | -1.135 | 0.0002 | Yes |
Table: GSEA details [plain text format]

  

Fig 2: DOROSZKO\_U3180MG\_CLUSTER\_4: Random ES distribution      
 Gene set null distribution of ES for **DOROSZKO\_U3180MG\_CLUSTER\_4**
